# Low-Level Laser Therapy (LLLT) in Dystrophin-Deficient Muscle Cells: Effects on Regeneration Capacity, Inflammation Response and Oxidative Stress

**DOI:** 10.1371/journal.pone.0128567

**Published:** 2015-06-17

**Authors:** Aline Barbosa Macedo, Luis Henrique Rapucci Moraes, Daniela Sayuri Mizobuti, Aline Reis Fogaça, Fernanda dos Santos Rapucci Moraes, Tulio de Almeida Hermes, Adriana Pertille, Elaine Minatel

**Affiliations:** 1 Departamento de Biologia Estrutural e Funcional, Instituto de Biologia, Universidade Estadual de Campinas (UNICAMP), Campinas, SP, Brazil; 2 Graduate Program in Science of Human Movement, Universidade Metodista de Piracicaba (UNIMEP), Piracicaba, SP, Brazil; University of Pittsburgh, UNITED STATES

## Abstract

The present study evaluated low-level laser therapy (LLLT) effects on some physiological pathways that may lead to muscle damage or regeneration capacity in dystrophin-deficient muscle cells of *mdx* mice, the experimental model of Duchenne muscular dystrophy (DMD). Primary cultures of *mdx* skeletal muscle cells were irradiated only one time with laser and analyzed after 24 and 48 hours. The LLLT parameter used was 830 nm wavelengths at 5 J/cm² fluence. The following groups were set up: Ctrl (untreated C57BL/10 primary muscle cells), *mdx* (untreated *mdx* primary muscle cells), *mdx* LA 24 (*mdx* primary muscle cells - LLLT irradiated and analyzed after 24 h), and *mdx* LA 48 (*mdx* primary muscle cells - LLLT irradiated and analyzed after 48 h). The *mdx* LA 24 and *mdx* LA 48 groups showed significant increase in cell proliferation, higher diameter in muscle cells and decreased MyoD levels compared to the *mdx* group. The *mdx* LA 48 group showed significant increase in Myosin Heavy Chain levels compared to the untreated *mdx* and *mdx* LA 24 groups. The *mdx* LA 24 and *mdx* LA 48 groups showed significant increase in [Ca^^2+^^]i. The *mdx* group showed significant increase in H_2_O_2_ production and 4-HNE levels compared to the Ctrl group and LLLT treatment reduced this increase. GSH levels and GPx, GR and SOD activities increased in the *mdx* group. Laser treatment reduced the GSH levels and GR and SOD activities in dystrophic muscle cells. The *mdx* group showed significant increase in the TNF-α and NF-κB levels, which in turn was reduced by the LLLT treatment. Together, these results suggest that the laser treatment improved regenerative capacity and decreased inflammatory response and oxidative stress in dystrophic muscle cells, indicating that LLLT could be a helpful alternative therapy to be associated with other treatment for dystrophinopathies.

## Introduction

Duchenne muscular dystrophy (DMD) is a degenerative and progressive muscular disease caused by the absence of the dystrophin protein, affecting about 1 in 3,500 male births and for which there is no effective therapy [1]. Corticosteroids are currently the standard treatment prescribed to DMD patients, but their benefits are modest and they have numerous side effects [2].

Abnormal reactive oxygen species (ROS) level and exaggerated inflammatory process contribute strongly to pathological mechanisms triggered by the absence of dystrophin in DMD [3–7]. Elevated levels of nuclear factor kappa-B (NF-κB), a transcription factor that regulates the expression of pro-inflammatory cytokines [3], and tumor necrosis factor alpha (TNF-α), a key cytokine that stimulates the inflammatory response [4] are found in circulating blood and in skeletal muscles in DMD patients and *mdx* mice, the experimental model of DM [5–7]. In addition, dystrophic muscle fibers of DMD patients and *mdx* mice display high levels of oxidative stress markers and lipid peroxidation by-products [6, 8–10].

Low-level laser therapy (LLLT) has been used in the treatment of inflammatory diseases, mainly in musculoskeletal disorders such as tendinopathies [11] and muscle injuries [12]. This therapy shows modulatory effects on NF-κB, TNF-α, IL-1β and COX-2 inflammatory markers [13–15]; reduces the inflammatory process itself (e.g. edema and hemorrhagic formation) [16]; and modulates leucocyte activity [17]. LLLT has also demonstrated favorable effects in modulating the oxidative stress by decreased ROS [18] and increased activity of antioxidant enzymes such as superoxide dismutase (SOD), catalase (CAT) and glutathione peroxidase (GPx) [19].

Considering that LLLT shows potential effects on oxidative stress and inflammatory response and also exhibits an advantage over the pharmacological therapy by not having severe side effects [12], we evaluated the effects of LLLT therapy on some physiological pathways that may lead to muscle damage or regeneration capacity in the dystrophin-deficient muscle cells of *mdx* mice. By using the *in vitro* cell culture of dystrophic fibers, we were able to better evaluate ROS production and inflammatory cytokines by dystrophin-deficient fibers per se specifically, without the interference of other cells normally present in the whole tissue, such as macrophages that contribute to H_2_O_2_ and TNF-α production.

## Materials and Methods

### Cell cultures

C57BL/10 mice (C57BL/10ScCr/PasUnib) and *mdx* mice (C57BL/10-Dmdmdx/PasUnib) were housed in animal house of State University of Campinas (UNICAMP), with food and water being available *ad libitum*. The animal experiments described here were conducted in accordance with the guidelines of the Brazilian College for Animal Experimentation and the guidelines set forth by our institution. The protocol (#2974–1) was approved by the Committee on the Ethics of Animal Experiments of UNICAMP, São Paulo, Brazil. Primary culture of skeletal muscle cells (PCSMC) was performed following the method described by Rando and Blau [20]. Male and female from *mdx* and C57BL/10 mice were sacrificed at 28 days of age by decapitation. The quadriceps femoris, tibialis anterior, extensor digitorum longus, gastrocnemius, soleus, and plantaris muscles were removed and used to prepare primary muscle culture. Muscles were triturated using a pair of scissors and enzymatically digested with collagenase and trypsin solutions at 37°C. The satellite cells (5x10^4^ cells/cm^2^) were plated in 1% Matrigel-coated dishes. The primary muscle cells were cultured in a proliferation and growth medium containing DMEM with glucose (5.5 mM), L-glutamine (2 mM), fetal bovine serum (10% v/v), horse serum (10% v/v), and penicillin/streptomycin (1% v/v) for 2 days. Myogenesis (myotube differentiation) was induced by the addition of a fusion medium (FM) that consisted of DMEM with glucose (5.5 mM), Lglutamine (2 mM), and horse serum (10% v/v). The culture was maintained at 37°C and 5% CO2 and the differentiated muscle cells with contractile properties were observed at 6–8 days of culture in the FM. Skeletal muscle cell cultures at 6–8 days were used in all experiments and all measurements were obtained from triplicate cultures. The following groups were studied:(1) Ctrl (primary muscle cells from C57BL/10 mice that did not receive any treatment), (2) *mdx* untreated (primary muscle cells from *mdx* mice that did not receive any treatment), (3) *mdx* LA 24 (primary muscle cells from *mdx* mice that received irradiation one time alone with LLLT and analyzed after 24 h), and (4) *mdx* LA 48 (primary muscle cells from *mdx* mice that received irradiation one time alone with LLLT and analyzed after 48 h).

### Low-level laser therapy

The experiments were conducted with the Aluminum Gallium Arsenide (AIGaAs) diode, 830 nm wavelength at 5 J/cm² fluence, continuous emission during 20 seconds, 30 mW output power and 0.07 cm^2^ beam area (IBRAMED laserpulse). PCSMC were irradiated one time alone with laser and analyzed after 24 and 48 hours. Immediately before of irradiation, the cell culture medium was replaced to phosphate-bufferd saline (PBS) to avoid loss of laser energy through absorption by colored culture medium [21]. Furthermore, the beam was positioned perpendicularly at 1 cm of lower surface plate and irradiation was performed by one point at the center of each culture well, without moving the laser tip. During these procedures the room was remained without light.

### PCSMC Analysis

Morphological characteristics of the PCSMC were investigated and compared by inverted microscope (Nikon, Eclipse TS100/TS100F) during the experimental period. Primary muscle cells diameter was quantified by measuring a total of 100 tube diameters from ten random fields at 20× magnification using the ImagePro-Express software (Media Cybernetic, Silver Spring, MD, USA). All data are expressed as the mean ± SD.

### MTT Assay for PCSMC Proliferation

The metabolic activity of cells was assessed by tetrazolium [3-(4,5-dimethylthiazol-2-yl)-2,5-diphenyltetrazolium bromide] (MTT; Sigma) assay. Bioreduction of tetrazolium salts probably reflects the integrated pyridine nucleotide dependent redox state of the cell. Therefore, the amount of formazan product is proportional to the metabolic activity of cells in culture and provides a widely used, although indirect, measurement of cell proliferation [22]. Briefly, PCSMC were washed in PBS, treated with MTT solution (5 mg/ml, tetrazolium salt) and incubated for 4h at 37°C. After 4 h, the cell supernatants were discarded, MTT crystals were dissolved with acid isopropanol and the absorbance measured at 570 nm. Plates were analyzed in a multi-mode microplate reader model Synergy H1M (Bio Tek Instruments, Washington) at 570 nm with a 655 nm reference wave length to quantify the amount of formazan product, which reflects the number of cells in culture. Wells that did not contain cells were used as a zero point of absorbance. All assays were performed in triplicate.

### Intracellular calcium content

For qualitative and quantitative measurements of intracellular calcium concentrations, [Ca^2+^]i, cells were loaded with calcium-sensitive dye Fluo-4 (Molecular Probes, Oregon, USA). PCSMC were loaded with the dye Fluo-4 AM for 60 min at room temperature at a concentration of 1 mmol/L (plus 0.005% Pluronic F-127 (Invitrogen, Oregon, USA)), as described previously [23]. The intensities of fluo-4 fluorescence was monitored on a fluorescent inverted microscope (Nikon, Eclipse TS100/TS100F), at excitation and emission wavelengths of 494 and 516 nm, respectively.

### Determination of H_2_O_2_ Production in PCSMC

Skeletal muscle cells were maintained in phenol red-free culture medium and muscle-derived ROS were determined using a fluorescence assay. The Amplex UltraRed reagent (50 μM) and HRP (0.1 U/ml) were added for 60 min. Amplex reacts with H_2_O_2,_ in the presence of HRP, to produce resorufin, a red fluorescent and stable compound. The fluorescence signal of resorufin was determined at 530 (excitation) and 590 nm wavelength (emission). Measurements of ROS were previously calibrated using exogenous 10 μM H_2_O_2_ (positive control). All measurements were performed in phenol red-free culture medium (1 ml), pH 7.4, at 37°C.

### Western blotting

Proteins were extracted in a buffer containing Tris–HCl (100 mM), pH 7.5; EDTA (10 mM), pH 8.0; sodium pyrophosphate (10 mM); sodium fluoride (0.1 mM); sodium orthovanadate (10 mM); PMSF (2 mM); and aprotinin (10 μg/ml). The cell extracts were sonicated for 30 s at 4°C. The homogenates were centrifuged at 11,000 g for 20 min at 4°C and the supernatants were treated with Triton X-100 (1%) and transferred to a −80°C freezer until being used for Western blotting analysis. An aliquot from the supernatant was used to determine the total protein content by the Bradford method. Thirty μg of total protein homogenate was loaded on 6%–15% SDS-polyacrylamide gels. Proteins were transferred from the gels to a nitrocellulose membrane using a submersion electrotransfer apparatus (Bio-Rad Laboratories, Hercules, California). Membranes were blocked for 2 h at room temperature with 5% skim milk/Tris-HCl buffer saline-Tween buffer (TBST; 10 mM Tris-HCl, pH 8, 150 mM NaCl, and 0.05% Tween 20). The membranes were incubated with the primary antibodies overnight at 4°C, washed in TBST, incubated with the peroxidase-conjugated secondary antibodies for 2 h at room temperature, and developed using the SuperSignal West Pico Chemiluminescent Substrate kit (Pierce Biotechnology, Rockford, Illinois). To control protein loading, Western blot transfer, and nonspecific changes in protein levels, the blots were stripped and reprobed for glyceraldehyde-3-phosphate dehydrogenase (GAPDH). Band intensities were quantified using ImageJ 1.38X (National Institutes of Health, Bethesda, Maryland) software. The following primary antibodies were used for Western blotting: (1) Dystrophin (mouse monoclal, Vector Laboratories, Ontario, Canadá); (2) NF-κB (goat polyclonal, Santa Cruz Biotechnology, Santa Cruz, California); (3) TNF-α (rabbit anti-mouse polyclonal, Chemical, USA); (4) 4-HNE (goat polyclonal, Santa Cruz Biotechnology, Santa Cruz, California); (5) MyoD (rabbit polyclonal, Santa Cruz Biotechnology, Santa Cruz, California); (6) Anti-Skeletal Myosin (mouse monoclonal, Sigma; Saint Louis, USA); (7) glyceraldehyde-3-phosphate dehydrogenase (GAPDH; rabbit polyclonal, Santa Cruz Biotechnology, Santa Cruz, California). The secondary antibody used was peroxidase-labeled affinity purified mouse, anti-goat or rabbit IgG antibody (KPL).

### Superoxide dismutase activity (SOD)

SOD activity was analyzed by the reduction of nitroblue tetrazolium using a Xanthine–Xanthine oxidase system, that is, superoxide generation [24]. The results were expressed as SOD units per mg of protein.

### Glutathione (GSH) content

Total GSH content was determined by Ellman's reaction using 5′5′-dithio-bis-2-nitrobenzoic acid (DTNB) as described by Anderson [25]. The intensity of the yellow color was read at 412 nm. The results were expressed as nmol per mg of protein.

### Glutathione peroxidase activity (GPx)

GPx activity was quantified by following the decrease in absorbance at 365 nm induced by 0.25 mM H2O2 in the presence of reduced glutathione (10 mM), NADPH, (4 mM), and 1 U enzymatic activity of GR [26]. Results were expressed as nmol per min per mg of protein.

### Glutathione reductase activity (GR)

GR activity was measured according to Carlberg and Mannervick [27], following the decrease in absorbance at 340 nm induced by oxidized glutathione in the presence of NADPH in phosphate buffer, pH 7.8. Absorbance changes were read between 1 and 10 min. Results were expressed as nmol per min per mg of protein.

### Statistical Analysis

All data are expressed as mean ± standard deviation (SD). Statistical analysis for direct comparison between means of groups was performed by ANOVA one way, followed by Bonferroni test used for multiple statistical comparisons between groups. P≤0.05 was considered statistically significant.

## Results

### Morphology and cell proliferation in control and dystrophic primary muscle cells

**Fig 1:**
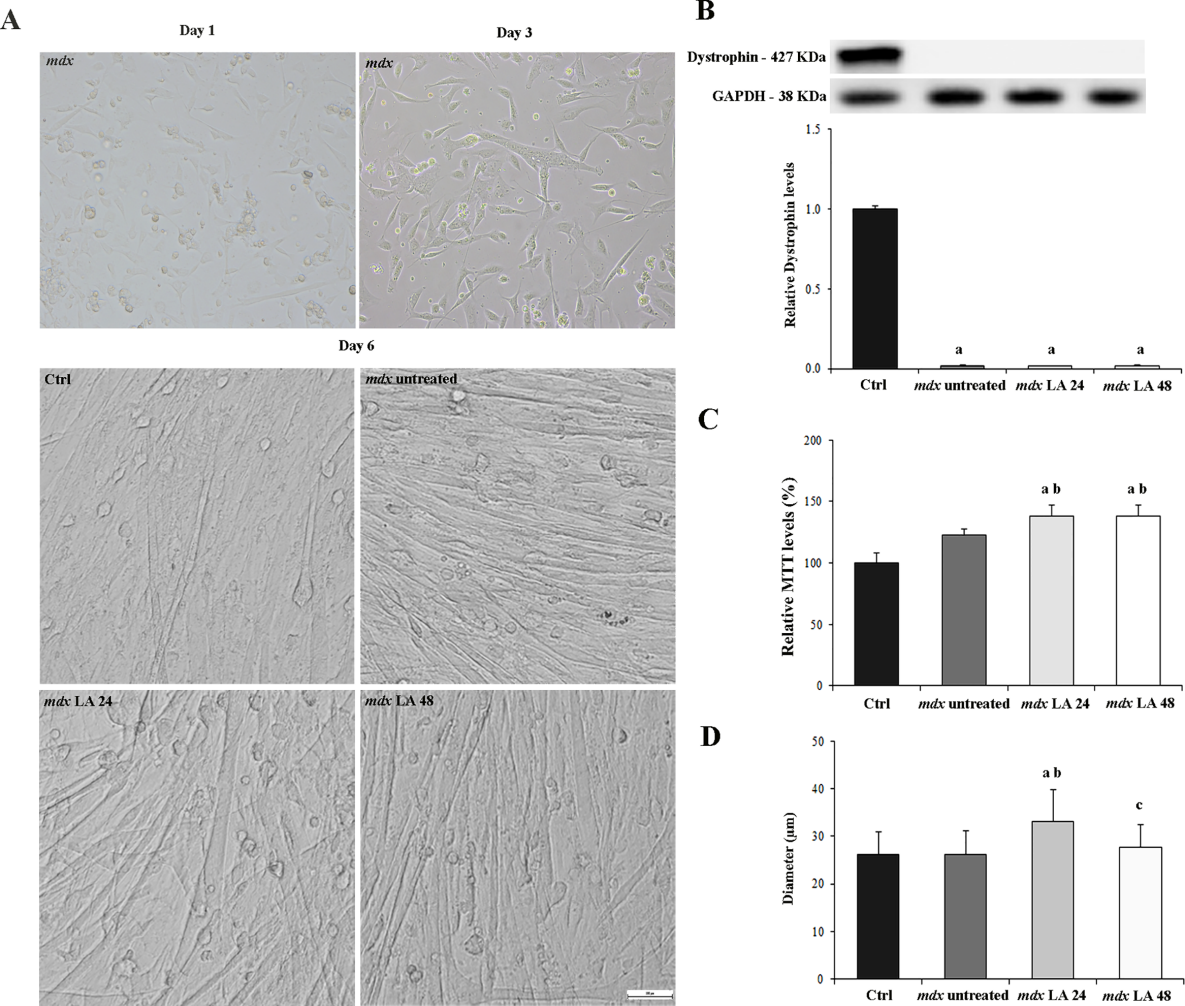
Morphology and cell proliferation in control and dystrophic muscle cells. In (A), morphology of normal (Ctrl) and dystrophic primary muscle cultures untreated (*mdx* untreated) and LLLT treatment analyzed after 24 hours (*mdx* LA 24) and 48 hours (*mdx* LA 48). Day 1 shows undifferentiated dystrophin-deficient (*mdx*) muscle cells; day 3 shows maturation process in *mdx* muscle cells and day 6 shows complete morphological maturation with organized sarcomeric structures. In (B), immunoblot analysis of dystrophin and graph showing protein level in the primary muscle cells from Ctrl, *mdx* untreated, *mdx* LA 24 and *mdx* LA 48. Glyceraldehyde-3-phosphate dehydrogenase (GAPDH) was used as a loading control. In (C), cell proliferation was assessed by measurement of MTT assay in the primary muscle cells from Ctrl, *mdx* untreated, *mdx* LA 24 and *mdx* LA 48. In (D), diameter myotubes from Ctrl, *mdx* untreated, *mdx* LA 24 and *mdx* LA 48. All the experiments were performed in triplicate, and the relative value of the band intensity was quantified and normalized by the corresponding Ctrl. ^a^ P< 0.05 versus Ctrl; ^b^ P< 0.05 versus *mdx* untreated; ^c^ P< 0.05 versus *mdx* LA 24. Error bars, SD.

Day 1 shows undifferentiated dystrophin-deficient (*mdx*) muscle cells; day 3 shows maturation process in *mdx* muscle cells and day 6 shows complete morphological maturation with organized sarcomeric structures (Fig 1A). PCSMC from normal (C57BL/10) and *mdx* primary muscle cells showed a similar time-course development, characterized by typical progression of proliferation to differentiation and fusion into thick, branching myotubes (Fig 1A). Contractile fibers were observed at 7 days in both cultures. The presence or absence of dystrophin was verified using western blots (Fig 1B). The significant difference in cell proliferation, as evaluated by MTT assay, was observed between the control and the *mdx* untreated cultures (Fig 1C). Likewise, the *mdx* LA 24 and *mdx* LA 48 groups showed significant increase (by 15% for both groups) in cell proliferation compared to *mdx* untreated cultures. Morphological quantification by the diameter of the primary muscle cells from control and *mdx* groups proved that all cells were following a growth pattern (Fig 1D). Laser-treated-dystrophic primary muscle cells (*mdx* LA 24 group) showed greater diameter (Ctrl: 26 μm; *mdx* untreated: 26 μm; *mdx* LA 24: 33 μm; *mdx* LA 48: 28 μm) compared to other experimental groups (Fig 1D).

### Myogenic regulatory factors and Myosin Heavy Chain in control and dystrophic primary muscle cells

**Fig 2:**
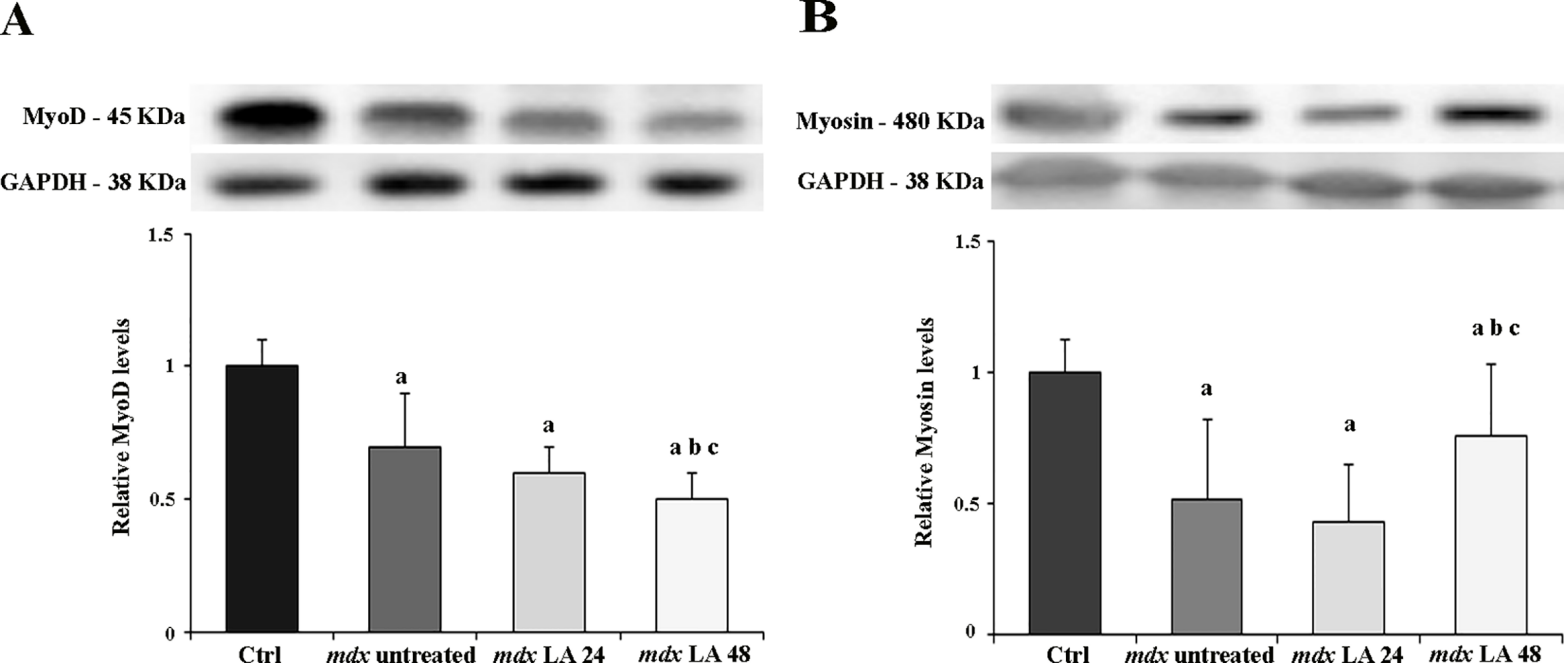
MyoD and Myosin Heavy Chain analysis in control and dystrophic muscle cells. Immunoblot analysis of MyoD (A) and Myosin Heavy Chain (B) and graphs showing protein level in the primary muscle cells from normal (Ctrl) and dystrophic primary muscle cells untreated (*mdx* untreated) and LLLT treatment analyzed after 24 hours (*mdx* LA 24) and 48 hours (*mdx* LA 48). Glyceraldehyde-3-phosphate dehydrogenase (GAPDH) was used as a loading control. All the experiments were performed in triplicate, and the relative value of the band intensity was quantified and normalized by the corresponding Ctrl. ^a^ P< 0.05 versus Ctrl; ^b^ P< 0.05 versus *mdx* untreated; ^c^ P< 0.05 versus *mdx* LA 24. Error bars, SD.

A number of reports have shown that the use of laser therapy is associated with the promotion of myogenesis. So, in this study we also evaluated the MyoD levels (Fig 2A) and Myosin Heavy Chain levels (MyHC) (Fig 2B). The primary muscle cells showed a reduction of MyoD levels for *mdx* (by 46%), *mdx* LA 24 (by 58%) and *mdx* LA 48 (by 80%) groups compared to the control. Also, it was observed that the *mdx* LA 48 group showed a reduction in the MyoD levels by 34% and 22% in relation to the untreated *mdx* and *mdx* LA 24 groups, respectively. The primary muscle cells showed a reduction of Myosin Heavy Chain levels for *mdx* (by 48%), *mdx* LA 24 (by 56%) and *mdx* LA 48 (by 24%) groups compared to the control. The *mdx* LA 48 group showed a increase in the Myosin Heavy Chain levels by 32% and 43% in relation to the untreated and *mdx* LA 24 groups, respectively.

### Intracellular calcium concentrations in control and dystrophic primary muscle cells

**Fig 3:**
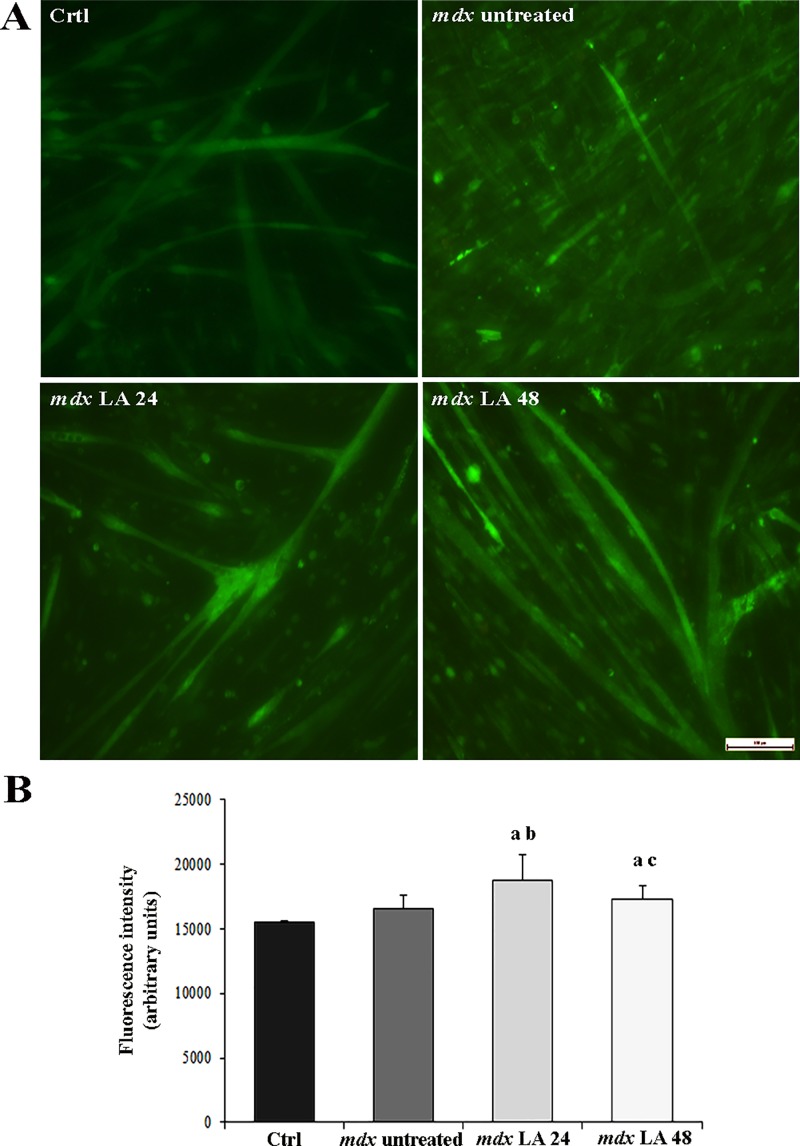
Intracellular calcium concentrations in control and dystrophic muscle cells. In (A), intracellular calcium concentrations, [Ca^2+^]i, was assessed by measurement of calcium-sensitive dye Fluo-4 (green) in the primary muscle cells from normal (Ctrl) and dystrophic primary muscle cells cultures untreated (*mdx* untreated) and LLLT treatment analyzed after 24 hours (*mdx* LA 24) and 48 hours (*mdx* LA 48). In (B), graph showing fluorescence intensity of [Ca^2+^]i in the primary muscle cells from Ctrl, *mdx* untreated, *mdx* LA 24 and *mdx* LA 48. All the experiments were performed in triplicate and data expressed as mean ± SD. ^a^ P< 0.05 versus Ctrl; ^b^ P< 0.05 versus *mdx* untreated; ^c^ P< 0.05 versus *mdx* LA 24.

A significant difference in [Ca^2+^]i was found by comparing dystrophic-laser and control primary muscle cells (Fig 3A and 3B). The *mdx* LA 24 and *mdx* LA 48 groups showed a significant increase in [Ca^2+^]i by 20% and 11%, respectively, compared to the Ctrl group (Fig 3B). In addition, the *mdx* LA 24 group showed higher [Ca^2+^]i (13% and 9%, respectively) compared to *mdx* untreated and *mdx* LA 48 groups.

### Analysis of oxidative stress in control and dystrophic primary muscle cells

**Fig 4:**
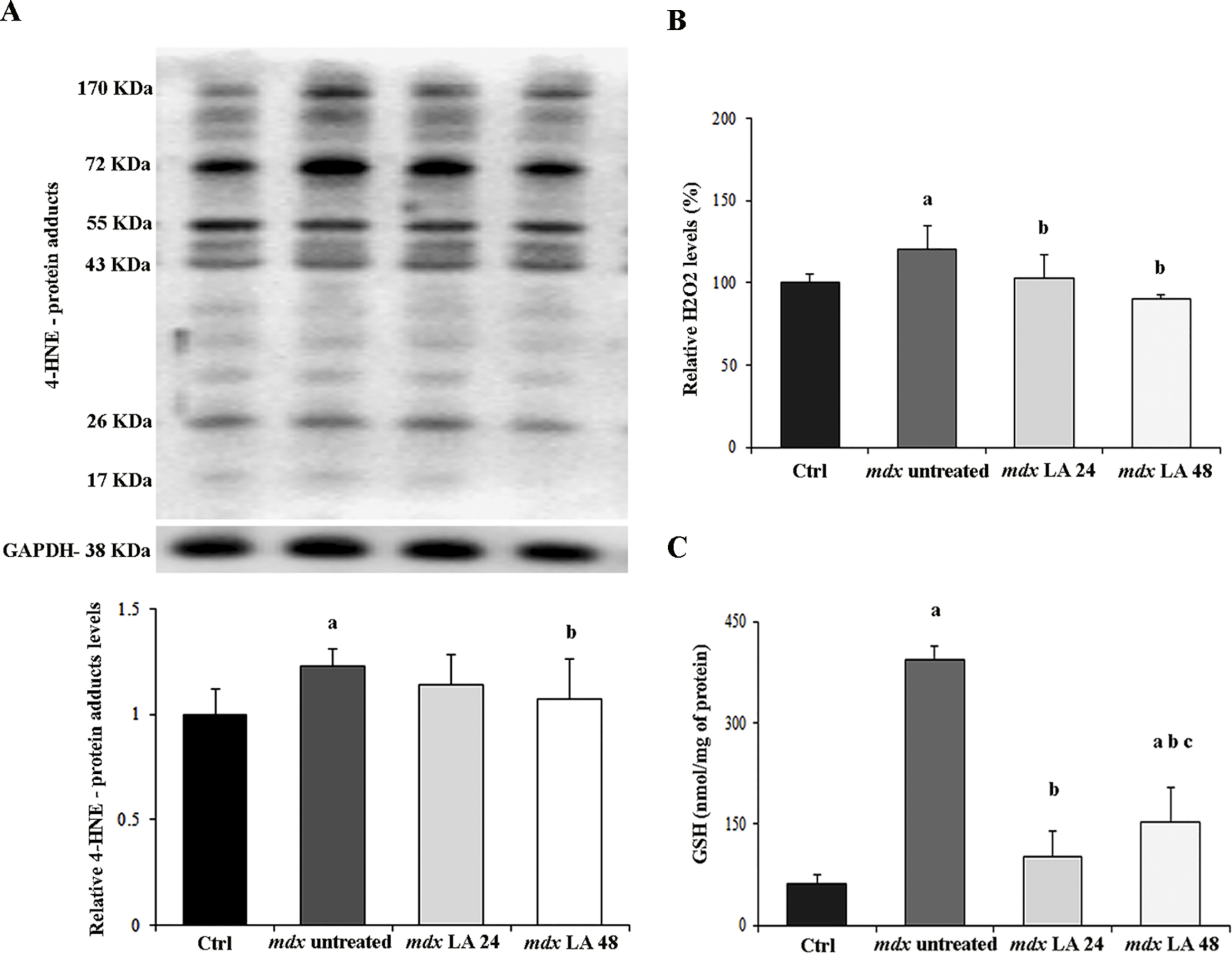
Analysis of oxidative stress in control and dystrophic muscle cells. In (A), immunoblot analysis shows several bands of 4-HNE-protein adducts, ranging from 17 to 170 kDa. Graphs show protein level in the muscle cells from Ctrl, *mdx* untreated, *mdx* LA 24 and *mdx* LA 48. Glyceraldehyde-3-phosphate dehydrogenase (GAPDH) was used as a loading control. In (B), quantification of H_2_O_2_ production in the muscle cells from normal (Ctrl) and dystrophic primary muscle cells untreated (*mdx* untreated) and LLLT treatment analyzed after 24 hours (*mdx* LA 24) and 48 hours (*mdx* LA 48). In (C) analysis of glutathione levels in the muscle cells from normal (Ctrl) and dystrophic culture cells untreated (*mdx* untreated) and LLLT treatment analyzed after 24 hours (*mdx* LA 24) and 48 hours (*mdx* LA 48). All the experiments were performed in triplicate and data expressed as mean ± SD. The relative value of the band intensity was quantified and normalized by the corresponding Ctrl. ^a^ P< 0.05 versus Ctrl; ^b^ P< 0.05 versus *mdx* untreated; ^c^ P< 0.05 versus *mdx* LA 24.

In order to analyze the laser effects on oxidative stress in dystrophic primary muscle cells, we determined 4-hydroxynonenal (4-HNE)-protein adduct levels; H_2_O_2_ production; glutathione (GSH) content and superoxide dismutase (SOD); glutathione peroxidase (GPx); and glutathione reductase (GR) activity. Bands of 4-HNE-protein adducts are shown in Fig 4A. Proteins from 17 to 170 kDa were observed in all groups. The 4-HNE protein adduct levels were significantly higher in *mdx* primary muscle cells (by 19%) compared to control primary muscle cells (P<0.05; Fig 4A). Reduction of 13% on the 4-HNE protein adduct levels was observed in the *mdx* LA 48 group (P<0.05; Fig 4A). *Mdx* primary muscle cells showed a significant increase in H_2_O_2_ production (by 20%) compared to control primary muscle cells (Fig 4B). Treatment with laser significantly decreased the H_2_O_2_ production (by 17% for *mdx* LA 24 and 30% for *mdx* LA 48) in dystrophic primary muscle cells (Fig 4B). GSH levels were significantly higher in *mdx* primary muscle cells (by six times) compared to control primary muscle cells (P<0.05; Fig 4C). LLLT treatment significantly decreased the GSH levels (by 73% for *mdx* LA 24 and 60% for *mdx* LA 48) in dystrophic primary muscle cells (Fig 4C). The effect of the laser treatment on SOD, GPx and GR activities is shown in Table 1. The increase of SOD, GPx and GR activities in *mdx* group were found to be significant when compared to the Ctrl group (P<0.05; Table 1). LLLT treatment significantly decreased the GR (by 42% for *mdx* LA 24) and SOD activity (by 47% for *mdx* LA 24 and 32% for *mdx* LA 48) in dystrophic primary muscle cells (Table 1).

**Table 1:** SOD, GPx and GR enzimatic activity in control and dystrophic muscle cells.

Group	SOD (SOD/mg protein)	GPx (nmol/min/mg of protein)	GR (nmol/min/mg of protein)
**Ctrl**	24.04±1.45	8.4±5.16	15.0±4.62
***mdx* untreated**	515.42±10.6 ^a^	118.6±29.29 ^a^	249.5±19.81 ^a^
***mdx* LA 24**	271.7±4.03 ^a^ ^b^	132.1±14.47 ^a^	144.2±48.20 ^a^ ^b^
***mdx* LA 48**	346.98±7.81 ^a^ ^b^ ^c^	144.6±27 .39 ^a^	248.90±49.43 ^a^ ^c^

Analysis of superoxide dismutase (SOD), glutathione peroxidase (GPx) and glutathione reductase activity (GR) in the primary muscle cells from normal (Ctrl) and dystrophic untreated (*mdx* untreated) and LLLT treatment analyzed after 24 hours (*mdx* LA 24) and 48 hours (*mdx* LA 48). All the experiments were performed in triplicate and data expressed as mean ± SD.

^a^ P< 0.05 vs Ctrl group.

^b^ P< 0.05 vs *mdx* untreated group.

^c^ P< 0.05 vs *mdx* LA 24 group.

### Analysis of inflammation in control and dystrophic primary muscle cells

**Fig 5:**
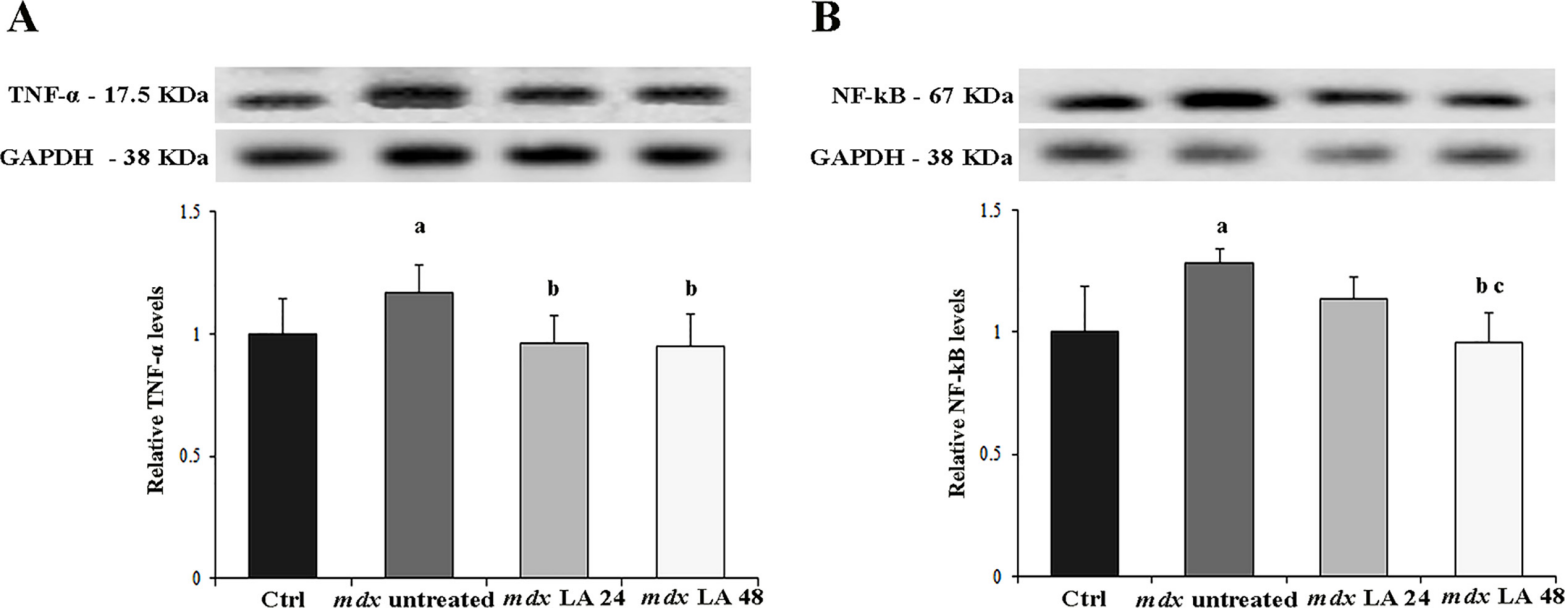
Analysis of inflammation in control and dystrophic muscle cells. Immunoblot analysis of TNF-α (A) and NF-κB (B). Graphs show protein level in the muscle cells from normal (Ctrl) and dystrophic primary muscle cells untreated (*mdx* untreated) and LLLT treatment analyzed after 24 hours (*mdx* LA 24) and 48 hours (*mdx* LA 48). Glyceraldehyde-3-phosphate dehydrogenase (GAPDH) was used as a loading control. All the experiments were performed in triplicate, and the relative value of the band intensity was quantified and normalized by the corresponding Ctrl. ^a^ P< 0.05 versus Ctrl; ^b^ P< 0.05 versus *mdx* untreated; ^c^ P< 0.05 versus *mdx* LA 24. Error bars, SD.

Inflammatory response of laser therapy was analyzed by the TNF-α and NF-κB levels (Fig 5A and 5B). Dystrophic primary muscle cells presented high levels of TNF-α and NF-κB (by 15% and 22%, respectively) compared to the control. The *mdx* LA 24 and *mdx* LA 48 primary muscle cells showed a significant reduction (by 8% and 9%, respectively) on TNF-α levels compared to control. In addition, the *mdx* LA 48 group showed a significant decrease on NF-κB levels (by 25% and 14%, respectively) compared to Ctrl and *mdx* LA 24 groups.

## Discussion

A variety of lasers with different wavelengths and biological effects are widely used to improve the repair of muscle damage [12]. In this study, we evaluated the effect of AIGaAs laser irradiation on the some physiological pathways that may lead to muscle damage or regeneration capacity in dystrophic cell culture.

The understanding of LLLT effect in terms of proliferation and myogenic regulatory factors (MRFs) can provide an important insight regarding muscle regeneration capacity in dystrophic cells. Our findings showed that the laser therapy promoted muscle cell proliferation in dystrophic cells. Also the morphological examination of the dystrophic cells, treated for 24 hours, demonstrated a 27% increase in the mean myotube diameter compared to the *mdx* untreated cells. Furthermore, dystrophic cultures showed earlier differentiation compared to the control cultures, seen as an early decline in MyoD levels and in addition to this, laser therapy accentuated this down-grade. In addition, the reduction of MyoD levels was accompanied by a significant increase in MyHC level after LLLT 48, which leads us to suggest that the LLLT treatment accelerated the differentiation process in dystrophic muscle cells. Similar to our results, Yablonka-Reuveni and Anderson [28] reported that the satellite cells from *mdx* mice display accelerated differentiation in primary cultures, demonstrated by the reduction in the MyoD expression and by a proportionate increase in myogenic expression. In addition, Nakano and collaborators [29] showed that LLLT increased satellite cells and maintained the diameter of rat myofibrils submitted to muscle atrophy process. Also, studies have shown that laser therapy affects satellite cell proliferation and differentiation *in vitro* [30, 31].

Deregulation of calcium concentration has been suggested to be an important factor involved in the pathogenesis of DMD [32]. While some investigators have reported elevated Ca^2+^ levels in DMD and *mdx* primary muscle cells [32, 33], others have not observed this increase [34–36]. Our results indicate that [Ca^2+^]i did not significantly alter in the dystrophin-deficient muscle cells. However, laser therapy promoted an increase in [Ca^2+^]i, particularly in the *mdx* LA 24 group. Researchers have shown that the high [Ca^2+^]i is one of the first events observed after laser therapy in several types of cells [37, 38].

Considering that accumulated ROS levels have an important role in the dystrophic muscle damage [39, 40], we also analyzed the LLLT effects on H_2_O_2_ and 4-HNE-protein adduct levels (a lipid peroxidation biomarker) in dystrophic muscle cells. Our results are not in agreement with some of those of previous studies. Lubart and collaborators [41] showed that laser therapy produces elevated ROS levels. In contrast, our data demonstrated that the LLLT exhibited antioxidant capacity by reducing the H_2_O_2_ and 4-HNE-protein adduct levels in dystrophic muscle cells. Another study also found that the laser therapy decreased oxidative stress markers, such as protein carbonyls and malondialdehyde (MDA) in DMD patients [42], which is consistent with our results. Huang and collaborators [43] also verified that laser therapy reduces oxidative stress in *in vitro* experiments and suggest that the LLLT mechanism interferes in the ROS concentration as it promotes changes in the mitochondrial membrane potential. Another possible explanation for the antioxidant properties of LLLT would be the up-regulation of the cellular antioxidant glutathione (GSH). In this study, we analyzed GSH and its associated regulatory enzymes (glutathione peroxidase- GPx and glutathione reductase-GR). At the same time, we also evaluated the SOD activity. The data obtained from GSH levels and GPx, GR and SOD activity suggest that the protective effects of LLLT could not be due to up-regulation of the antioxidant defense system. We suggest the laser acted directly as a scavenger of superoxide anions independent the enzymatic antioxidant system. This hypothesis is supported by work of Lim et al. [44], which showed that LLLT treatment can help remove the intracellular superoxide anion in SOD-inactivated cells, resulting in a decrease in lipid peroxidation.

Elevated ROS levels may be important in the activation of inflammatory pathways in the *mdx* muscle [45] and LLLT is also widely used to modulate the inflammatory process [46–48]. Our results showed that laser therapy has a pronounced anti-inflammatory effect in dystrophic muscle cells, by reducing the NF-kB and TNF-α levels. In agreement with our results, an *in vivo* study found that LLLT decreased the levels of several inflammatory markers, such as TNF-α, IL-6 and COX-2 in the dystrophic muscle of *mdx* mice [49]. In addition, this work demonstrates that the inflammatory response reduction was accompanied by a decrease in dystrophic muscle damage [49]. Another study also showed the protective effects of LLLT against muscle damage during postnatal development in hindlimb muscles of dystrophic mice [50]. Although the researchers did not clarify the LLLT mechanisms involved in this result, they suggest it may be associated with anti-inflammatory processes, enhanced angiogenesis and regeneration in dystrophic muscle [50].

In conclusion, our results, based on the source and parameters chosen, provide insights into potential applications of LLLT in promoting cell proliferation and lessening oxidative stress and inflammation process in dystrophin-deficient muscle cells. Although further studies are needed in order to clarify the LLLT action mechanisms in dystrophic muscle cells, we suggest that LLLT could be a helpful alternative therapy to be associated with other treatment for dystrophinopathies.
